# An Artificial Intelligence Model to Predict the Mortality of COVID-19 Patients at Hospital Admission Time Using Routine Blood Samples: Development and Validation of an Ensemble Model

**DOI:** 10.2196/25442

**Published:** 2020-12-23

**Authors:** Hoon Ko, Heewon Chung, Wu Seong Kang, Chul Park, Do Wan Kim, Seong Eun Kim, Chi Ryang Chung, Ryoung Eun Ko, Hooseok Lee, Jae Ho Seo, Tae-Young Choi, Rafael Jaimes, Kyung Won Kim, Jinseok Lee

**Affiliations:** 1 Biomedical Engineering Wonkwang University Iksan Republic of Korea; 2 Department of Trauma Surgery Wonkwang University Hospital Iksan Republic of Korea; 3 Department of Internal Medicine Wonkwang University Hospital Iksan Republic of Korea; 4 Department of Thoracic and Cardiovascular Surgery Chonnam National University Medical School Gwangju Republic of Korea; 5 Department of Internal Medicine Chonnam National University Medical School Gwangju Republic of Korea; 6 Department of Critical Care Medicine Samsung Medical Center Sungkyunkwan University School of Medicine Seoul Republic of Korea; 7 Department of Biochemistry Wonkwang University School of Medicine Iksan Republic of Korea; 8 Department of Pathology Wonkwang University School of Medicine Iksan Republic of Korea; 9 Biotechnology and Human Systems Lincoln Laboratory Massachusetts Institute of Technology Lexington, MA United States; 10 Radiology and Research Institute of Radiology Asan Medical Center University of Ulsan College of Medicine Seoul Republic of Korea

**Keywords:** COVID-19, artificial intelligence, blood samples, mortality prediction

## Abstract

**Background:**

COVID-19, which is accompanied by acute respiratory distress, multiple organ failure, and death, has spread worldwide much faster than previously thought. However, at present, it has limited treatments.

**Objective:**

To overcome this issue, we developed an artificial intelligence (AI) model of COVID-19, named EDRnet (ensemble learning model based on deep neural network and random forest models), to predict in-hospital mortality using a routine blood sample at the time of hospital admission.

**Methods:**

We selected 28 blood biomarkers and used the age and gender information of patients as model inputs. To improve the mortality prediction, we adopted an ensemble approach combining deep neural network and random forest models. We trained our model with a database of blood samples from 361 COVID-19 patients in Wuhan, China, and applied it to 106 COVID-19 patients in three Korean medical institutions.

**Results:**

In the testing data sets, EDRnet provided high sensitivity (100%), specificity (91%), and accuracy (92%). To extend the number of patient data points, we developed a web application (BeatCOVID19) where anyone can access the model to predict mortality and can register his or her own blood laboratory results.

**Conclusions:**

Our new AI model, EDRnet, accurately predicts the mortality rate for COVID-19. It is publicly available and aims to help health care providers fight COVID-19 and improve patients’ outcomes.

## Introduction

COVID-19 is a highly contagious infection caused by SARS-CoV2. In severe cases, COVID-19 causes acute respiratory distress, multiple organ failure, and, eventually, death [1]. As of November 2020, COVID-19 cases and deaths are approaching 60 million and 1.5 million, respectively, worldwide.

In a pandemic situation, the most important issue in the management of patients diagnosed with COVID-19 is to select patients at risk of high mortality in the early period of disease and to provide appropriate treatments [2]. Particularly, the condition of patients at high risk can rapidly deteriorate. Some papers reported that deceased COVID-19 patients initially had mild symptoms but suddenly transitioned to a critical stage, leading to death [3-5]. In Italy, 75% of deceased patients showed mild symptoms, such as fever, dyspnea, and cough, at admission to the hospital [1]. Thus, the development of a prognostic model to predict mortality as early as possible is very critical.

In this pandemic crisis, the shortage of resources and medical staff causes big problems in the health care system. Accordingly, artificial intelligence (AI) can aid in the management of COVID-19 patients. A recent research study has developed an AI prediction model of mortality based on blood test results [6]. In this study, Yan et al initially considered 73 blood-borne markers for the mortality prediction model; finally, three blood biomarkers were selected, including lactate dehydrogenase (LDH), lymphocyte, and high-sensitivity C-reactive protein (hs-CRP). This model predicted mortality with 90% accuracy based on a decision tree using an XGBoost classifier [7] to analyze feature importance.

However, Yan et al’s study has drawbacks. First, the three biomarkers derived from the XGBoost-based feature selection may not be the best choices. Feature importance provides a score indicating how each feature contributes in the construction of decision trees within the model. However, due to the stochastic nature of machine learning algorithms, each feature’s importance score may vary. Moreover, in decision tree algorithms, such as an XGBoost and a random forest (RF), when multiple features have the same gain during the split, a branch in a tree is made by randomly selecting features among them. Second, numerous studies have shown that the disease progression of COVID-19 is not only associated with LDH [2,8-11], lymphocyte [12,13], and hs-CRP [2,10,14-17] but also with other blood-based biomarkers, such as neutrophil counts [16,18,19], albumin [18,20,21], and prothrombin activity [18,22-24]. In our study, we developed an AI model using 28 biomarkers for predicting the mortality of COVID-19 patients. Third, the three biomarker-based AI models [6] predicted mortality 10 days before a patient’s recovery or death. These limitations show that the model may not work for COVID-19 patients who have just been diagnosed and hospitalized.

Therefore, in this study, we aimed to develop an AI model based on a blood test for mortality prediction at the early stage of hospital admission. We deployed the developed AI model on a public website so that all patients and medical staff could predict mortality using individual patient blood test results.

## Methods

### Data Sets

This study was approved by Wonkwang University Hospital (WKUH), Chonnam National University Hospital (CNUH), and Samsung Medical Center (SMC) in Korea. Informed consent was waived. For training data, we used the blood test results obtained from 375 COVID-19 patients collected between January 10, 2020, and February 24, 2020, in Tongji Hospital, Wuhan, China [6]. Of these, 14 patients without a blood test within 1 day after the hospital admission were excluded, and 361 patients—212 males (58.7%) and 149 females (41.3%); mean age 58.9 years (SD 16.5)—were included. As presented in Multimedia Appendix 1, the training data set of 361 patients included the admission date and time, discharge date and time, age, gender, mortality outcome, and results of blood tests obtained within 24 hours after hospital admission. For testing data, we collected medical records on COVID-19 patients (N=106) from three medical institutions: CNUH (85/106, 80.2%), WKUH (11/106, 10.4%), and SMC (10/106, 9.4%). The blood laboratory results from these 106 COVID-19 patients were collected between February 2020 and July 2020. Similar to the training data, we used the blood test data obtained within 24 hours after hospital admission (see Multimedia Appendix 2). For summarizing the statistics of the training and testing data sets, the patients were classified into a survivor group and a deceased group in the training and testing data sets. The number of blood tests differed across patients and institutions. The mean numbers of blood tests per patient were 61.21 (range 24-73) in the training data set and 35.36 (range 30-55) in the testing data set. The mean numbers of hospitalization days were 13.82 (survivor group) and 8.16 (deceased group) in the training data set and 18.21 (survivor group) and 17.98 (deceased group) in the testing data set (see Table 1).

**Table 1:** Statistical summary of the training and testing data sets.

Patient data		Training data set (N=361)	Testing data set (N=106)			
		Tongji Hospital	CNUH^a^ (n=85)	WKUH^b^ (n=11)	SMC^c^ (n=10)	Total (N=106)
**Number of patients, n (%)**						
	Total	361 (100)	85 (100)	11 (100)	10 (100)	106 (100)
	Survived	195 (54.0)	85 (100)	9 (82)	10 (100)	104 (98.1)
	Deceased	212 (58.7)	0 (0)	2 (18)	0 (0)	2 (1.9)
**Gender, n (%)**						
	Male	212 (58.7)	34 (40)	5 (45)	3 (30)	42 (39.6)
	Female	149 (41.3)	51 (60)	6 (55)	7 (70)	64 (60.4)
**Number of hospitalization days, mean (SD)**						
	Survived	13.82 (6.38)	15.06 (7.90)	28.16 (11.13)	30.95 (23.03)	18.21 (11.46)
	Deceased	8.16 (7.38)	N/A^d^	17.98 (11.83)	N/A	17.98 (11.83)
Age in years, mean (SD)		58.91 (16.49)	44.14 (21.81)	56.27 (23.00)	58.20 (21.05)	46.73 (22.28)
**Number of blood biomarkers collected**						
	Min-max	24-73	32-55	30-52	30-40	30-55
	Mean (SD)	61.21 (6.92)	36.89 (4.11)	35.00 (5.31)	34.20 (3.16)	35.36 (4.19)

^a^CNUH: Chonnam National University Hospital.

^b^WKUH: Wonkwang University Hospital.

^c^SMC: Samsung Medical Center.

^d^N/A: not applicable; there were no deceased patients in the testing data set at this institution.

### Feature Selection

Given the total 73 blood biomarkers from the training data, we performed an analysis of variance (ANOVA), which uses an *F* test to check for any significant difference between the two groups (ie, deceased vs survivor) according to each blood biomarker. For the feature selection, we also considered the available data rate (ADR), which refers to how much blood biomarker data were available for training the AI model. This is calculated as

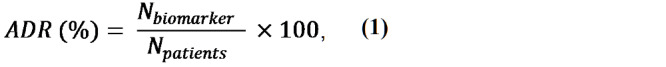



where *N_patients_* is the total number of patients (N=361) and *N_biomarker_* is the number of patients having each of the specific biomarker data.

Based on the ANOVA, we first selected the top 32 biomarkers corresponding to *P* values less than 10^–5^. Subsequently, we excluded four biomarkers with ADR values of less than 90%. Table 2 summarizes the final selection of 28 biomarkers with the corresponding ANOVA *P* values and ADR values. The ANOVA *P* values and ADR values for all 73 biomarkers in the training data set are summarized in Multimedia Appendix 3, Table S1. The sample distributions of the selected 28 biomarkers in the survivor and deceased groups are presented in Multimedia Appendix 3, Figure S1.

**Table 2:** List of 28 blood biomarkers selected for the artificial intelligence model training.

Biomarker index No.	Blood biomarker	ANOVA^a^ *P* value	ADR^b^, %
1	Lymphocytes	2.44×10^−46^	96.95
2	Neutrophils	5.65×10^−43^	96.68
3	Albumin	2.90×10^−37^	96.12
4	Lactate dehydrogenase	4.18×10^−36^	96.12
5	Neutrophil count	3.54×10^−35^	96.68
6	Hypersensitive C-reactive protein	8.38×10^−35^	94.74
7	Prothrombin activity	3.20×10^−26^	94.18
8	Calcium	2.24×10^−19^	95.29
9	Urea	3.29×10^−17^	96.12
10	Estimated glomerular filtration rate	5.05×10^−17^	96.12
11	Monocytes	1.09×10^−14^	96.95
12	Globulin	6.06×10^−13^	96.12
13	Eosinophils	2.07×10^−12^	96.68
14	Glucose	2.39×10^−11^	93.63
15	Red blood cell distribution width (RDW)	8.43×10^−10^	92.24
16	HCO_3_^−^ (bicarbonate)	2.68×10^−9^	96.12
17	RDW standard deviation	3.06×10^−9^	92.24
18	Platelet count	1.46×10^−8^	96.68
19	Mean platelet volume	1.92×10^−7^	92.24
20	Platelet large-cell ratio	2.02×10^−7^	92.24
21	Prothrombin time	3.42×10^−7^	94.18
22	Total protein	5.29×10^−7^	96.12
23	Platelet distribution width	6.98×10^−7^	92.24
24	Aspartate aminotransferase	1.01×10^−6^	96.12
25	Thrombocytocrit	1.49×10^−6^	92.24
26	Eosinophil count	2.90×10^−6^	92.24
27	Alkaline phosphatase	8.27×10^−6^	96.12
28	International standard ratio	2.65×10^−5^	92.24

^a^ANOVA: analysis of variance.

^b^ADR: available data rate.

### Preprocessing

Given the selected 28 biomarkers, the mean number of available biomarkers per patient was 27.22 (SD 2.33) for the training data and 16.86 (SD 1.58) for the testing data, as summarized in Table 3. To handle the missing data, we calculated the mean value from the training data for each biomarker and replaced the missing data with the mean value for the training and testing data sets. We then added two more features (ie, age and gender) to the 28 biomarkers and trained our AI model using 30 features.

**Table 3:** Number of available blood biomarkers per patient for the artificial intelligence model training.

Data sets and sources		Number of blood biomarkers			
		Mean (SD)		Min-max	
Training data: Tongji Hospital		27.22 (2.33)		13-28	
**Testing data**					
	Chonnam National University Hospital		20.39 (1.13)		19-24
	Wonkwang University Hospital		15.82 (1.94)		14-19
	Samsung Medical Center		14.40 (1.58)		14-17
	Total		16.86 (1.58)		14-24

With the 30 features, we performed data set standardization, which is a common requirement for machine learning estimators. The standardization changes the data distribution of each feature with zero mean and standard deviation of 1 as

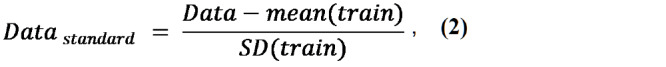



where *mean(train)* and *SD(train)* are the mean and standard deviation values, respectively, for each feature from the training data. The standardization was applied to the training and testing data sets.

### Development of an Ensemble AI Model

**Figure 1:**
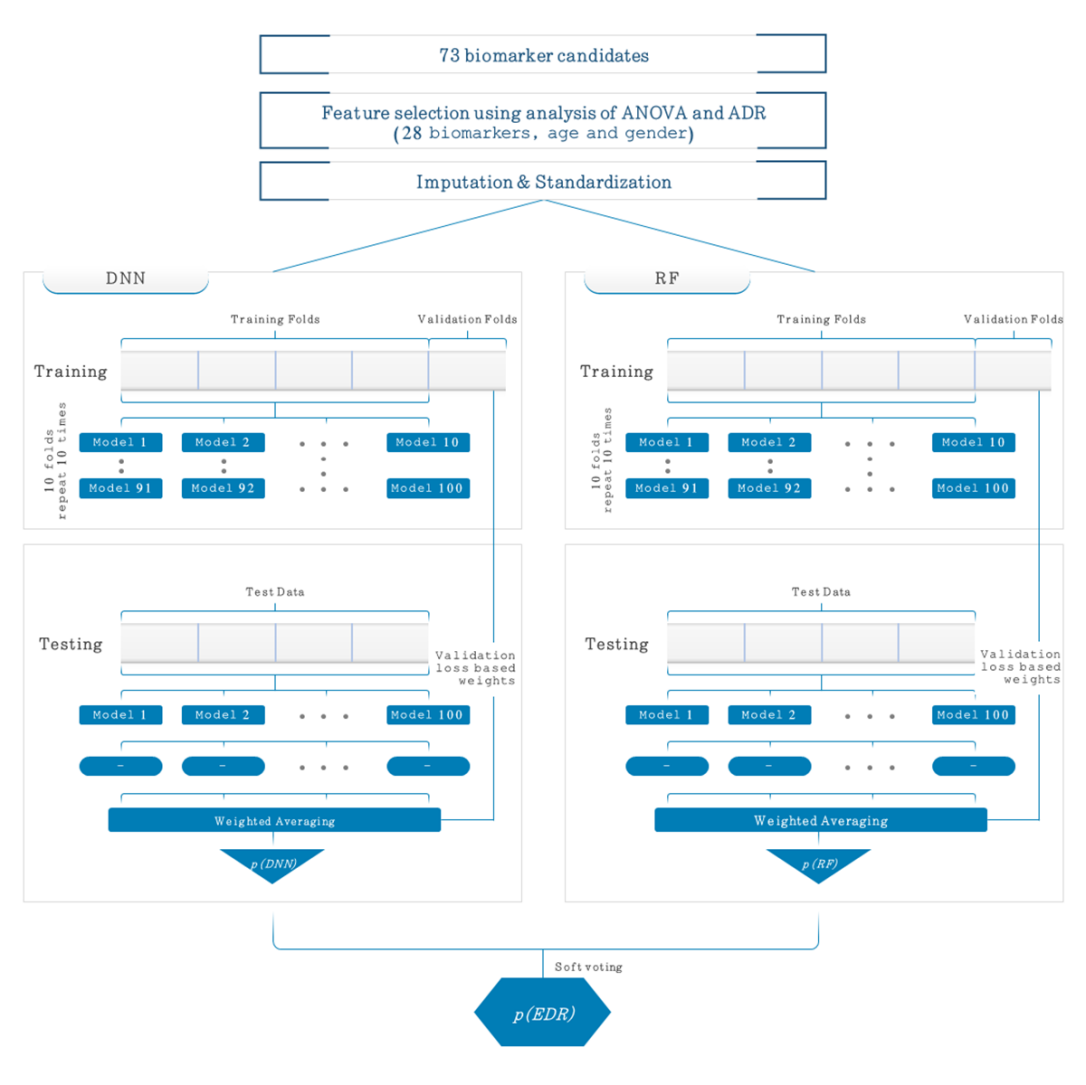
Proposed ensemble model (EDRnet) composed of a 5-layer deep neural network (DNN) and random forest (RF) model for the mortality prediction. In the training of both models, a 10-time-repetition 10-fold stratified cross-validation was separately performed, and the predicted mortality probabilities of the DNN model, p(DNN), and the RF model, p(RF), were calculated. The final predicted mortality probability of the ensemble model, p(EDR), was obtained by soft voting based on the p(DNN) and the p(RF). ADR: available data rate; ANOVA: analysis of variance; EDRnet: ensemble learning model based on DNN and RF models.

As illustrated in Figure 1, the new ensemble AI model is composed of a 5-layer deep neural network (DNN) and RF model. Our ensemble AI model was named as EDRnet (ensemble learning model based on DNN and RF models). The 5-layer DNN was comprised of an input layer, three fully connected (FC) layers, and an output layer. The input layer contained 30 features, including 28 biomarkers, age, and gender. The input layer was fed into three FC layers in a series, each of which consisted of 30, 16, and 8 nodes. To alleviate the overfitting issue, we applied a dropout rate of 0.3. Then, the last FC layer was fed into a softmax layer, which is an output layer providing the probabilities for the patient mortality. Figure S2 in Multimedia Appendix 3 shows our DNN model and its printed textual summary run on Keras, where the total number of parameters (ie, weights and biases) was 1571.

For the 5-layer DNN, a 10-time-repetition 10-fold stratified cross-validation was performed to confirm the model’s generalization ability. The training data (N=361) were randomly shuffled and partitioned into 10 equal subgroups in a stratified manner. Of the 10 subgroups, a single subgroup was retained as the validation data set for testing the model, and the remaining nine subgroups were used as the training data set. The process was then repeated 10 times, with each of the 10 subgroups used exactly once as the validation data set. By repeating this stratified 10-fold cross-validation process 10 times, a total of 100 models from the 5-layer DNN were derived. Then, we ensembled the models with the weighted average as

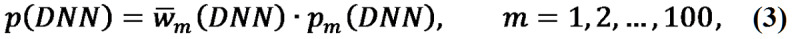



where *p_m_(DNN)* is the predicted mortality probability value from the *m*^th^ model of the DNN, *p(DNN)* is the ensemble result corresponding to the predicted mortality prediction probability, and 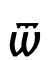
*_m_(DNN)* is the normalized weight value for the *m*^th^ model. We obtained the normalized weight value 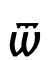
*_m_(DNN)* as

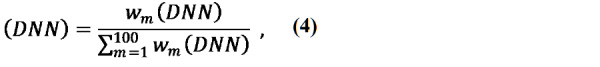



where the weight *w_m_(DNN)* was obtained using the validation loss from the *m*^th^ model, *l_m_(DNN)*, as

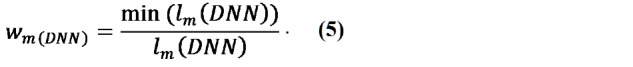



Along with the 5-layer DNN, we separately trained an RF model. For the RF model, 100 decision trees were trained with a maximum depth of 4 and maximum feature number of 5. Similar to the 5-layer DNN, we performed a 10-time-repetition 10-fold stratified cross-validation and ensembled the 100 models with the weighted average as

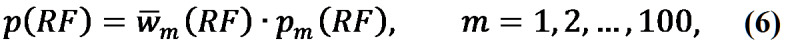



where *p_m_(RF)* is the predicted mortality probability value from the *m*^th^ model of the RF, *p(RF)* is the ensemble result corresponding to the predicted mortality prediction probability, and 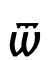
*_m_(RF)* is the normalized weight value for the *m*^th^ model. We obtained the normalized weight value 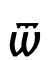
*_m_* as

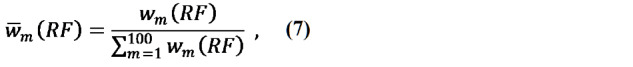



where the weight *w_m_(RF)* was obtained using the validation loss from the *m*^th^ model, *l_m_(RF)*, as

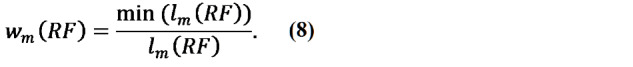



Given the two ensemble results *p(DNN)* and *p(RF),* we finally obtained the final predicted mortality probability value using soft voting. Based on the average of the two probability values *p(DNN)* and *p(RF),* if the value is greater than or equal to 0.5, then the prediction result represents death; otherwise, it represents survival.

### Implementation

We implemented and trained EDRnet using TensorFlow, version 1.13.1 for graphics processing unit (GPU), and Keras, version 2.2.4 for GPU. NumPy, version 1.16.4; Pandas, version: 0.25.3; Matplotlib, version 3.1.2; and scikit-learn, version 0.22.1, were used to build the model and analyze the results. We trained the models with the Adam optimizer and a binary cross-entropy cost function in equation 9 with a learning rate of 0.0001 and a batch size of 64 on the NVIDIA GeForce GTX 1080 Ti GPU as

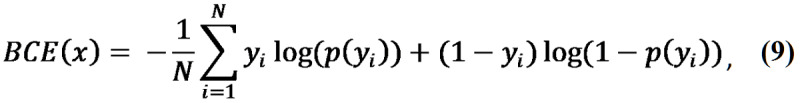



where *y_i_* is the label (ie, 1 for deceased and 0 for survived) and *p(y_i_)* is the predicted probability of each patient being deceased for the batch size *N* number of patients.

### Performance Evaluation of AI Models

To evaluate the performance of the AI models in predicting mortality, we used the sensitivity, specificity, accuracy, and balanced accuracy metrics, defined as

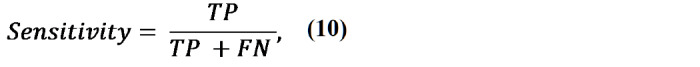

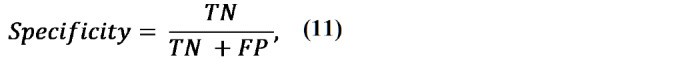

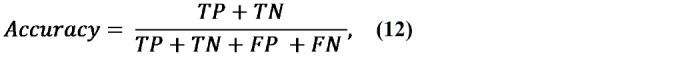

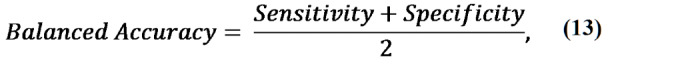









where TP, TN, FP, and FN represent the true positive, true negative, false positive, and false negative, respectively.

In the training data set, the prediction performance of the AI models was evaluated based on a 10-time-repetition 10-fold stratified cross-validation. In the testing data set, the prediction performance of the AI models was independently evaluated.

To compare the performance of our proposed EDRnet model with those of other external AI models, we separately trained the models of XGBoost and AdaBoost (AB), each of which was evaluated as a single model and as an ensemble model combined with DNN, resulting in four models: XGBoost, AB, ensemble with DNN and XGBoost (EDX), and ensemble with DNN and AB (EDA). For the training of these models, we searched the optimal hyperparameters providing the highest validation accuracy value, as presented in Multimedia Appendix 3, Table S2. In addition, we adopted a recently published AI model by Li et al [6] using a decision tree via an XGBoost-based feature selection for performance comparison. All five external AI models were evaluated using our testing data set of 106 patients.

## Results

The cross-validation of RF, DNN, and our ensemble model EDRnet showed that the accuracy on the validation data set is 89% for RF, 92% for DNN, and 93% for EDRnet. Thus, EDRnet provides the highest sensitivity, specificity, accuracy, and balanced accuracy values (see Table 4).

**Table 4:** Cross-validation accuracy comparison.

Model	Cross-validation results (N=361), mean (SD)			
	Sensitivity	Specificity	Accuracy	Balanced accuracy
Random forest	0.89 (0.06)	0.89 (0.07)	0.89 (0.04)	0.89 (0.04)
Deep neural network	0.91 (0.06)	0.93 (0.04)	0.92 (0.04)	0.92 (0.06)
EDRnet^a^	0.92 (0.05)	0.93 (0.03)	0.93 (0.03)	0.93 (0.05)

^a^EDRnet: ensemble learning model based on deep neural network and random forest models.

Moreover, we applied EDRnet to 106 Korean patients as an independent testing data set to check the TP, TN, FP, FN, sensitivity, specificity, accuracy, and balanced accuracy. The results show a sensitivity of 100%, specificity of 91%, accuracy of 92%, and balanced accuracy of 96%, indicating that the model trained and validated on Chinese patient data can be applied to Korean patients for mortality prediction (see Table 5). The computational times of DNN and RF in EDRnet for the training were 796 and 126 seconds, respectively. The overall computational time for the testing of EDRnet was 72 seconds.

**Table 5:** Test results from our proposed EDRnet (ensemble learning model based on deep neural network and random forest models) model.

Model	Testing data	True negative, %	False positive, %	False negative, %	True positive, %	Sensitivity	Specificity	Accuracy	Balanced accuracy
EDRnet	CNUH^a^	79	6	0	0	N/A^b^	0.93	0.93	0.93
EDRnet	WKUH^c^	7	2	0	2	1.00	0.78	0.82	0.89
EDRnet	SMC^d^	9	1	0	0	N/A	0.90	0.90	0.90
EDRnet	Total	95	9	0	2	1.00	0.91	0.92	0.96

^a^CNUH: Chonnam National University Hospital.

^b^N/A: not applicable.

^c^WKUH: Wonkwang University Hospital.

^d^SMC: Samsung Medical Center.

Next, we summarized the performance comparison results between XGBoost, AB, RF, DNN, EDX, EDA, Li et al’s model [6], and EDRnet. Considering all variables, EDRnet provided the highest prediction performance. Indeed, the balanced accuracy was 88% with XGBoost, 89% with AB, 92% with RF, 71% with DNN, 88% with EDX, 71% with EDA, 67% with Li et al’s model [6], and 96% with EDRnet. Notably, the accuracy of Li et al’s model was only 36%, indicating that a few blood markers may not be sufficient to predict patient mortality (see Table 6).

**Table 6:** Comparison of the performance of various methods.

Model	True negative, %	False positive, %	False negative, %	True positive, %	Sensitivity	Specificity	Accuracy	Balanced accuracy
XGBoost	80	24	0	2	1.00	0.77	0.77	0.88
AdaBoost	81	23	0	2	1.00	0.78	0.78	0.89
Random forest	87	17	0	2	1.00	0.84	0.84	0.92
5-layer deep neural network (DNN)	95	9	1	1	0.50	0.91	0.90	0.71
DNN + XGBoost	80	24	0	2	1.00	0.77	0.77	0.88
DNN + AdaBoost	96	8	1	1	0.50	0.92	0.91	0.71
Li et al’s model [6]	36	68	0	2	1.00	0.35	0.36	0.67
DNN + random forest (EDRnet^a^)	95	9	0	2	1.00	0.91	0.92	0.96

^a^EDRnet: ensemble learning model based on DNN and random forest models.

**Figure 2:**
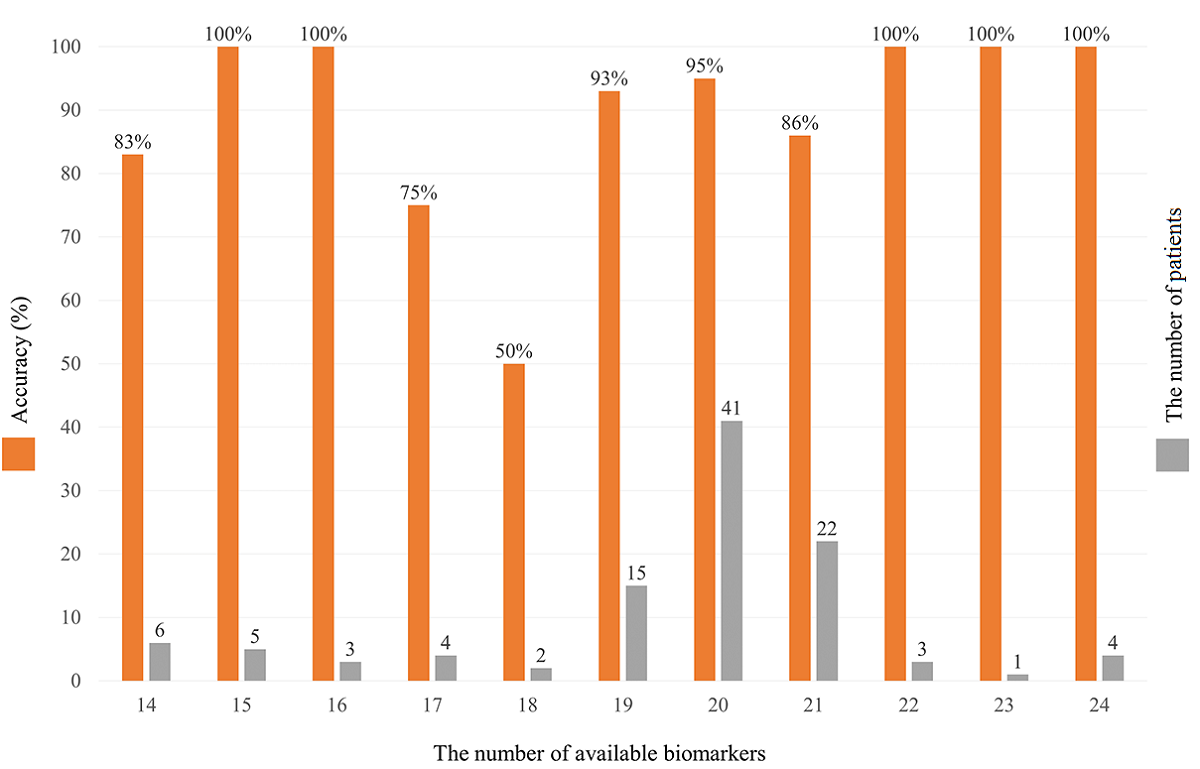
Accuracy with the number of available blood biomarkers from the 106-patient testing data set.

Our proposed EDRnet model used 28 blood biomarkers for prediction, but it does not require all 28 blood biomarkers. In our testing data sets, EDRnet training was validated using available biomarkers, ranging from 14 to 24, for each patient (see Figure 2). The results reveal that the majority of the patients had 19 to 21 available biomarkers (ie, 19 in 15 patients, 20 in 41 patients, and 21 in 22 patients) with a similarly high prediction accuracy (ie, 93%, 95%, and 86%, respectively). For the patients with 17 and 18 available biomarkers, the accuracy was 75% and 50%, respectively. By contrast, the patients with 14 to 16 biomarkers showed a high accuracy ranging from 83% to 100%.

**Figure 3:**
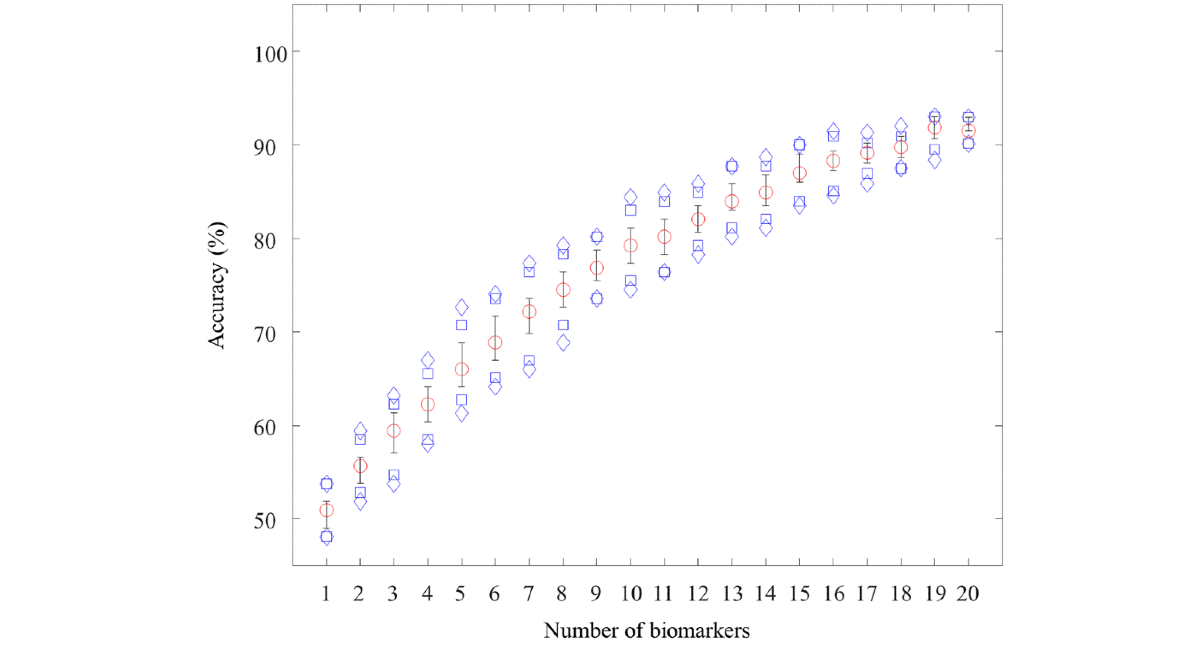
Estimated accuracy values according to the number of available biomarkers. Red circles represent the median. The bars at the top and bottom represent the 75th and 25th percentiles, respectively. The blue rectangles at the top and bottom represent the 90th and 10th percentiles, respectively. The blue diamonds at the top and bottom represent the 95th and 5th percentiles, respectively.

To further investigate the effect of the number of available biomarkers, we estimated the accuracy values according to the number of available biomarkers (see Figure 3). For the estimation, we randomly selected 1 to 20 biomarkers from all of the testing data points and tested the model with a 100-time repetition. When randomly selecting biomarkers, only samples where the actual available number of biomarkers was equal to or greater than the number of randomly selected biomarkers were simulated. The results show that accuracy increases with the number of available biomarkers until reaching 19 biomarkers.

**Figure 4:**
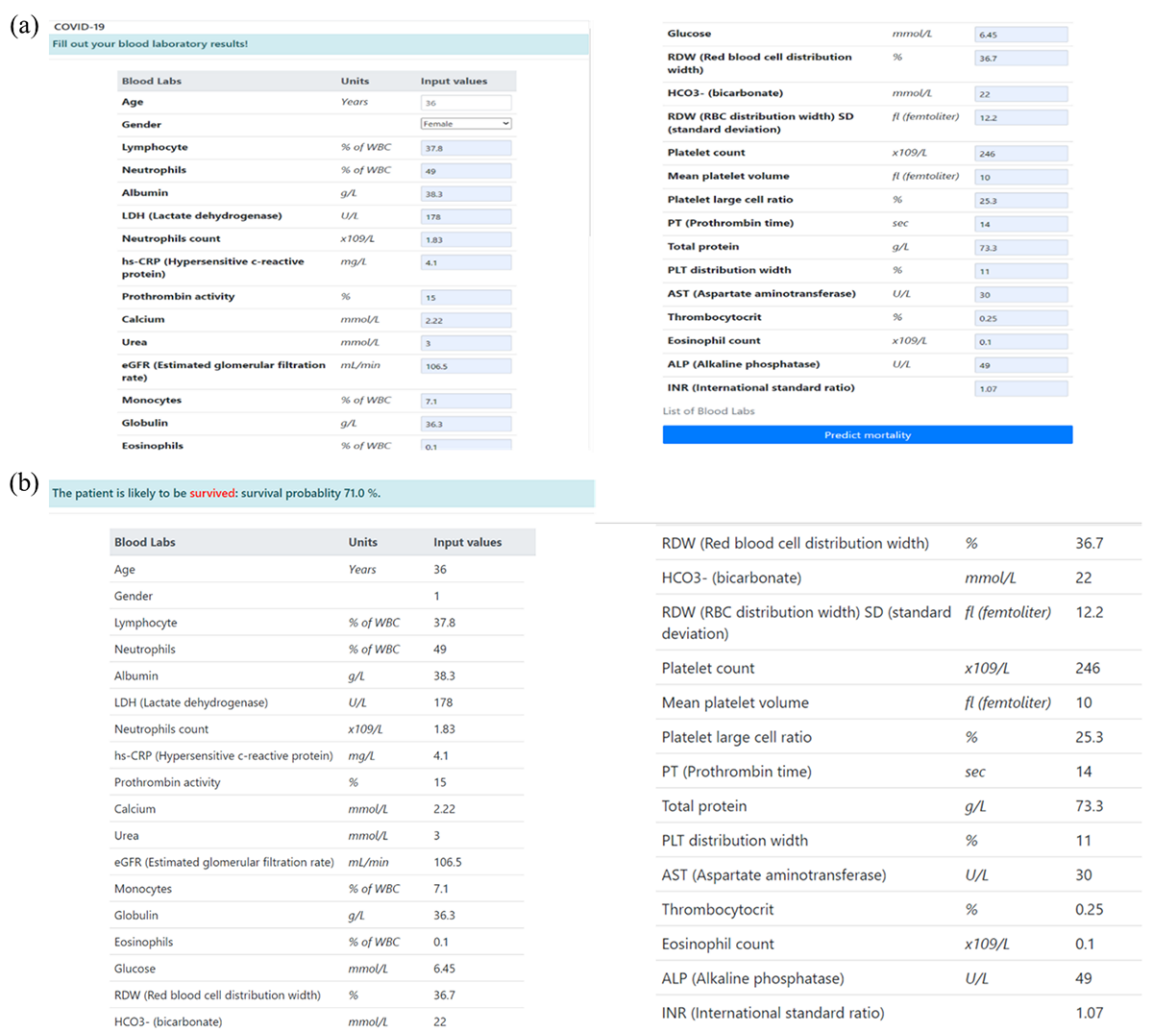
Deployed web application, BeatCOVID19 [25]: (a) input windows where a user inputs his or her blood sample results and (b) the predicted mortality results after entering the blood sample results.

Furthermore, our developed AI model, EDRnet, was successfully deployed on a public website [25] so that anyone can predict mortality using individual blood test results. The web application provides predicted mortality probability, as shown in Figure 4. A user inputs his or her blood sample results (see Figure 4a), and then the predicted mortality results are presented (see Figure 4b). Currently, the web application does not store any information entered by users. However, we consider and plan to store information entered by users on agreement to improve the AI model via a real-time learning process.

Regarding clinical characteristics (see Table 7), there were no significant differences in comorbidity. In terms of initial symptoms, the deceased group had more frequent dyspnea symptoms than the survivor group (66.7% vs 16.8%; *P*=.04). All patients from the deceased group required oxygen supply. The deceased group had more frequent altered mentality than the survivor group (50.0% vs 1.0%; *P*=.02). There was no significant difference in terms of antiviral drugs (ie, lopinavir or ritonavir, chloroquine or hydroxychloroquine, ribavirin, remdesivir, and oseltamivir) or anti-inflammatory drugs (ie, interferon, dexamethasone, and methylprednisolone) between the deceased and survivor groups. However, the deceased group received more antibiotics or combination therapy.

**Table 7:** Clinical characteristics of the patient groups from the testing data set.

Characteristics		Deceased group (n=2)	Survivor group (n=104)	Total (N=106)	*P* value
**Comorbidity, n (%)**					
	Diabetes mellitus	0 (0)	10 (9.6)	10 (9.4)	>.99
	Asthma	0 (0)	8 (7.7)	8 (7.5)	>.99
	Chronic obstructive pulmonary disease	0 (0)	0 (0)	0 (0)	>.99
	Coronary heart disease	0 (0)	3 (2.9)	3 (2.8)	>.99
	Cardiovascular disease	0 (0)	1 (1.0)	1 (0.9)	>.99
	Chronic kidney disease	0 (0)	1 (1.0)	1 (0.9)	>.99
	Chronic liver disease	0 (0)	0 (0)	0 (0)	>.99
	Congestive heart failure	1 (50)	3 (2.9)	4 (3.8)	.11
	Cancer	0 (0)	3 (2.9)	3 (2.8)	>.99
**Initial symptom, n (%)**					
	Fever	2 (100)	59 (56.7)	61 (57.5)	.61
	Cough	1 (50)	46 (44.2)	47 (44.3)	>.99
	Dyspnea	2 (100)	18 (16.8)	20 (18.2)	.04
	Diarrhea	0 (0)	11 (10.3)	11 (10.0)	>.99
	Myalgia	0 (0)	26 (24.3)	26 (23.6)	>.99
**Initial vital sign, mean (SD)**					
	Systolic blood pressure (mm Hg)	127.5 (17.7)	128.0 (18.5)	128.0 (18.4)	.97
	Diastolic blood pressure (mm Hg)	74.5 (10.6)	78.8 (12.8)	78.7 (12.8)	.64
	Heart rate (per minute)	96.5 (24.7)	84.2 (17.7)	84.4 (17.8)	.34
	Respiration rate (per minute)	29.5 (10.6)	20.2 (4.1)	20.4 (4.3)	.43
Altered mentality, n (%)		1 (50)	1 (1.0)	2 (1.9)	.02
**Oxygen requirement, n (%)**					
	No oxygen supply	0 (0)	83 (79.8)	83 (78.3)	.07
	Conventional oxygen	1 (50)	15 (14.4)	16 (15.1)	.69
	High-flow nasal cannula	0 (0)	3 (2.9)	3 (2.8)	>.99
	Noninvasive ventilation	0 (0)	0 (0)	0 (0)	>.99
	Mechanical ventilation	1 (50)	3 (2.9)	4 (3.8)	.11
	Extracorporeal membrane oxygenation	0 (0)	1 (1.0)	1 (0.9)	>.99
**Pharmacologic agent, n (%)**					
	Lopinavir or ritonavir	2 (100)	30 (28.8)	32 (30.2)	.16
	Chloroquine or hydroxychloroquine	0 (0)	7 (6.7)	7 (6.6)	>.99
	Ribavirin	0 (0)	0 (0)	0 (0)	>.99
	Remdesivir	0 (0)	0 (0)	0 (0)	.99
	Oseltamivir	0 (0)	2 (1.9)	2 (1.9)	.99
	Interferon	0 (0)	0 (0)	0 (0)	>.99
	Dexamethasone	0 (0)	1 (1.0)	1 (0.9)	>.99
	Methylprednisolone	0 (0)	4 (3.8)	4 (3.8)	>.99
	Antibiotics	2 (100)	8 (7.7)	10 (9.4)	.001
	Combination	2 (100)	15 (14.4)	17 (16.0)	.02

## Discussion

### Principal Findings

Our new AI model, EDRnet, was able to predict the mortality of COVID-19 patients using 28 blood biomarkers obtained within 24 hours after hospital admission. In the independent testing data sets, EDRnet showed excellent prediction performance with high sensitivity (100%), specificity (91%), and accuracy (92%). We were able to improve the prediction performance by adopting the ensemble approach combining DNN and RF models. Of note, EDRnet was developed by training with Chinese patients’ data and testing with Korean patients’ data.

EDRnet has several advantages. First, EDRnet can predict which patients are at a high risk of mortality in the early stage of hospital admission (ie, within 24 hours after admission). This is a substantial improvement compared to the prior AI prediction model reported by Yan et al, which predicted mortality 10 days before the occurrence of survival or death [6]. The mortality prediction at the time of admission can be substantially informative for clinicians because the critical time regarding disease progression is 10 to 14 days from the onset of symptoms, according to previous studies [13,16,26]. EDRnet can provide treatment priority guidance regarding who should be treated intensively. Second, EDRnet only uses blood biomarkers to predict mortality. In general, COVID-19 patients get blood laboratory tests at the time of hospital admission [9,27]. Blood biomarkers are objective indices that are used to estimate patients’ conditions in a quantitative manner, which may be beneficial to assure the reliability of the AI model. We did not include subjective biomarkers, such as symptoms, nor predisposing factors, such as underlying comorbidities, because these indices are difficult for quantification and may show high variability between patients. Third, the clinical meaning and significance of blood biomarkers used in our EDRnet model have been well investigated through many prior clinical studies. Thus, the AI’s predicted mortality results are explainable and easily understood by doctors. Furthermore, several major blood biomarkers are used in our EDRnet model.

The hematological changes in lymphocytes, neutrophils, monocytes, eosinophils, and platelets are common, as these changes are related to viral replication and hyperinflammation in COVID-19 infection [12,13]. In severe cases, the infiltration and sequestration of CD4+/CD8+ T cells occurred, leading to a decrease in the peripheral lymphocytes. Neutrophil counts [19-21] were significantly higher in the severe group than in the mild group. Platelet count, platelet volume, and platelet large-cell ratio are related to COVID-19 infection because immunologic destruction can lead to inappropriate platelet activation and consumption as well as impaired megakaryopoiesis [28-30].

Regarding blood chemistry, hs-CRP is a major biomarker that represents acute phase inflammation [2,10,14-17]. LDH is related to cell damage, so elevated LDH is an independent risk factor for the severity and mortality of COVID-19 [2,8-11]. Hypoalbuminemia [18,20,21], hypocalcemia [31-33], and elevated aspartate aminotransferase [18] are highly associated with severe COVID-19 infection requiring hospitalization in the intensive care unit. Urea and estimated glomerular filtration rate are important lab findings associated with an underlying chronic renal disease, which is a well-known predisposing factor of mortality [34]. In terms of the coagulation profile, COVID-19 generally presents a hypercoagulation state, thus resulting in an elevated prothrombin time and international normalized ratio in severe COVID-19 cases [3,18].

In this study, no significant differences were observed in the use of pharmacologic agents between the deceased and survivor groups except antibiotics and in the use of antiviral drugs, such as remdesivir. Antibiotics or combination therapy is usually used for suspected bacterial superinfection that represents severe diseases. To date, there has been no successfully effective pharmacologic agent to treat COVID-19. The pharmacologic treatment is not significantly related to survival in this study.

EDRnet does not require all 28 blood biomarkers for the prediction of mortality. EDRnet worked well as long as there were at least 19 blood biomarkers at the time of admission. Compared to prior AI prediction models for COVID-19 mortality, which used three biomarkers, there might be concern that EDRnet requires too many biomarkers. However, these blood tests are commonly performed in our daily clinical practice for hospitalized patients with COVID-19. If more data are accumulated, then we can reduce the number of blood biomarkers for mortality prediction.

### Limitations and Future Work

Our study has several limitations. First, the number of patients available for testing might be small. According to Johns Hopkins Coronavirus Resource Center, the mortality rate in South Korea is 1.7%. In the testing data set of 106 Korean patients, the mortality rate was 1.9%, which is almost equivalent to the actual mortality rate. It might be necessary to update EDRnet by training with a large population data set from all over the world. To update EDRnet, we made a web application [25] so that anyone can access the model. We believe that opening the AI model to the public is helpful to improve its performance and generalizability. Second, our data did not include other races, such as Caucasian or Middle East Asian. Our future research plan is to establish a real-time AI training system that can continue to train our model using prospectively collected data from all over the world. In addition, we will upgrade the web application so that the database framework allows a user to input his or her blood sample results along with the outcome. Based on the extended data, we will improve EDRnet for better generalization.

### Conclusions

In conclusion, our new AI model, EDRnet, was developed to predict the mortality of COVID-19 patients at the time of hospital admission using blood biomarkers only. It is now open to the public with the hope that it can help health care providers fight COVID-19 and improve patients’ outcomes.

## Appendix

Multimedia Appendix 1 Training data set.

Multimedia Appendix 2 Testing data set.

Multimedia Appendix 3 Supplementary figures and tables.

## Abbreviations

AB: AdaBoost
ADR: available data rate
AI: artificial intelligence
ANOVA: analysis of variance
CNUH: Chonnam National University Hospital
DNN: deep neural network
EDA: ensemble with deep neural network and AdaBoost
EDRnet: ensemble learning model based on deep neural network and random forest models
EDX: ensemble with deep neural network and XGBoost
FC: fully connected
FN: false negative
FP: false positive
GPU: graphics processing unit
hs-CRP: high-sensitivity C-reactive protein
LDH: lactate dehydrogenase
RF: random forest
SMC: Samsung Medical Center
TN: true negative
TP: true positive
WKUH: Wonkwang University Hospital
