# Supplementary material for: An Artificial Intelligence Model to Predict the Mortality of COVID-19 Patients at Hospital Admission Time Using Routine Blood Samples: Development and Validation of an Ensemble Model
Source: J Med Internet Res. 2020 Dec 23;22(12):e25442. doi: 10.2196/25442 (PMC7759509; doi:10.2196/25442)
Supplement: Multimedia Appendix 3 [file jmir_v22i12e25442_app3.docx]

Artificial intelligence can predict the mortality of COVID-19 patients at the admission time using routine blood samples

# Hoon Ko^1,^†, Heewon Chung^1,^†, Chul Park^2,^†, Do Wan Kim^3^, Seong Eun Kim^4^, Chi Ryang Chung^5^, Ryoung Eun Ko^5^, Hooseok Lee^1^, Jae Ho Seo^6^, Tae-Young Choi^7^, Rafael Jaimes^8^, Kyung Won Kim^9^, Wu Seong Kang,^10,^* and Jinseok Lee^1,^*

^1^Department of Biomedical Engineering, Wonkwang University School of Medicine, Iksan, Korea

^2^Department of Internal Medicine, Wonkwang University School of Medicine, Iksan, Korea

^3^Department of Thoracic and Cardiovascular Surgery, Chonnam National University Hospital, Chonnam National University Medical School, Gwangju, Korea

^4^Department of Internal Medicine, Chonnam National University Medical School, Gwangju, Korea

^5^Department of Critical Care Medicine, Samsung Medical Center, Sungkyunkwan University School of Medicine, Seoul 06351, Korea

^6^Department of Biochemistry, Wonkwang University School of Medicine, Iksan, Korea

^7^Department of Pathology, Wonkwang University School of Medicine, Iksan, Korea

^8^Biotechnology & Human Systems, Lincoln Laboratory, Massachusetts Institute of Technology, Lexington, MA, USA

^9^Radiology and Research Institute of Radiology, Asan Image Metrics, Clinical Trial Center, Asan Medical Center, University of Ulsan College of Medicine, Seoul, Republic of Korea

^10^Department of Trauma Surgery, Wonkwang University Hospital, Iksan, Republic of Korea

†These authors contributed equally to this work.

Table S1. ANOVA *p*-values and the available data rates for all 73 biomarkers (highlighted with 28 selected biomarkers)

| **Index** | **Blood biomarkers** | **ANOVA *p*-value** | **ADR (%)** |
| --- | --- | --- | --- |
| 1 | Lymphocyte | 2.44ⅹ10^-46^ | 96.95 |
| 2 | Neutrophils | 5.65ⅹ10^-43^ | 96.68 |
| 3 | Albumin | 2.90ⅹ10^-37^ | 96.12 |
| 4 | LDH (Lactate dehydrogenase) | 4.18ⅹ10^-36^ | 96.12 |
| 5 | Neutrophils count | 3.54ⅹ10^-35^ | 96.68 |
| 6 | hs-CRP (Hypersensitive c-reactive protein) | 8.38ⅹ10^-35^ | 94.74 |
| 7 | D dimer | 2.16ⅹ10^-26^ | 89.47 |
| 8 | Prothrombin activity | 3.20ⅹ10^-26^ | 94.18 |
| 9 | Calcium | 2.24ⅹ10^-19^ | 95.29 |
| 10 | Urea | 3.29ⅹ10^-17^ | 96.12 |
| 11 | eGFR (Estimated glomerular filtration rate) | 5.05ⅹ10^-17^ | 96.12 |
| 12 | Monocytes | 1.09ⅹ10^-14^ | 96.95 |
| 13 | FDP (Fibrin degradation products) | 4.08ⅹ10^-14^ | 45.71 |
| 14 | Globulin | 6.06ⅹ10^-13^ | 96.12 |
| 15 | Eosinophils | 2.07ⅹ10^-12^ | 96.68 |
| 16 | Glucose | 2.39ⅹ10^-11^ | 93.63 |
| 17 | RDW (Red blood cell distribution width) | 8.43ⅹ10^-10^ | 92.24 |
| 18 | HCO3- (bicarbonate) | 2.68ⅹ10^-9^ | 96.12 |
| 19 | RDW (RBC distribution width) SD (standard deviation) | 3.06ⅹ10^-9^ | 92.24 |
| 20 | Ferritin | 5.15ⅹ10^-9^ | 43.77 |
| 21 | Platelet count | 1.46ⅹ10^-8^ | 96.68 |
| 22 | Mean platelet volume | 1.92ⅹ10^-7^ | 92.24 |
| 23 | Platelet large cell ratio | 2.02ⅹ10^-7^ | 92.24 |
| 24 | PT (Prothrombin time) | 3.42ⅹ10^-7^ | 94.18 |
| 25 | Total protein | 5.29ⅹ10^-7^ | 96.12 |
| 26 | PLT distribution width | 6.98ⅹ10^-7^ | 92.24 |
| 27 | AST (Aspartate aminotransferase) | 1.01ⅹ10^-6^ | 96.12 |
| 28 | Thrombocytocrit | 1.49ⅹ10^-6^ | 92.24 |
| 29 | Eosinophil count | 2.90ⅹ10^-6^ | 96.68 |
| 30 | ALP (Alkaline phosphatase) | 8.27ⅹ10^-6^ | 96.12 |
| 31 | INR (International standard ratio) | 2.65ⅹ10^-5^ | 94.18 |
| 32 | Antithrombin | 4.40ⅹ10^-5^ | 45.71 |
| 33 | GGT (γ-glutamyl transpeptidase) | 1.21ⅹ10^-4^ | 96.12 |
| 34 | Potassium | 2.12ⅹ10^-4^ | 95.29 |
| 35 | NT-proBNP (Amino-terminal brain natriuretic peptide) | 2.59ⅹ10^-4^ | 62.60 |
| 36 | IL-2R (Interleukin 2 receptor) | 8.48ⅹ10^-4^ | 16.07 |
| 37 | Total bilirubin | 8.51ⅹ10^-4^ | 96.12 |
| 38 | Basophil count | 9.42ⅹ10^-4^ | 96.68 |
| 39 | Indirect bilirubin | 9.97ⅹ10^-4^ | 94.46 |
| 40 | Procalcitonin | 1.23ⅹ10^-3^ | 77.84 |
| 41 | Direct bilirubin | 1.25ⅹ10^-3^ | 96.12 |
| 42 | Basophil | 1.65ⅹ10^-3^ | 96.68 |
| 43 | IL-10 (Interleukin 10) | 1.86ⅹ10^-3^ | 16.07 |
| 44 | Uric acid | 2.32ⅹ10^-3^ | 96.12 |
| 45 | Lymphocyte count | 2.83ⅹ10^-3^ | 96.68 |
| 46 | IL-8 (Interleukin 8) | 3.07ⅹ10^-3^ | 16.07 |
| 47 | Total cholesterol | 3.46ⅹ10^-3^ | 96.12 |
| 48 | hs-TnI (Hypersensitive cardiac troponin I) | 5.46ⅹ10^-3^ | 68.98 |
| 49 | WBC (White blood cell) count | 6.01ⅹ10^-3^ | 97.23 |
| 50 | Corrected calcium | 6.61ⅹ10^-3^ | 95.29 |
| 51 | TT (Thrombin time) | 6.94ⅹ10^-3^ | 72.02 |
| 52 | ALT (Glutamic-pyruvic transaminase) | 7.13ⅹ10^-3^ | 96.12 |
| 53 | TNF-α (Tumor necrosis factor-α) | 8.58ⅹ10^-3^ | 16.07 |
| 54 | ESR (erythrocyte sedimentation rate) | 1.65ⅹ10^-2^ | 72.02 |
| 55 | HBsAg (hepatitis B surface antigen) | 4.18ⅹ10^-2^ | 62.33 |
| 56 | MCH (Mean corpuscular hemoglobin) | 4.49ⅹ10^-2^ | 96.68 |
| 57 | RBC (Red blood cell) count | 5.02ⅹ10^-2^ | 97.23 |
| 58 | MCHC (Mean corpuscular hemoglobin concentration) | 5.65ⅹ10^-2^ | 96.68 |
| 59 | IL-6 (Interleukin 6) | 5.75ⅹ10^-2^ | 17.17 |
| 60 | Creatinine | 1.62ⅹ10^-1^ | 96.12 |
| 61 | Sodium | 1.71ⅹ10^-1^ | 95.29 |
| 62 | HCV antibody quantification | 1.87ⅹ10^-1^ | 62.33 |
| 63 | MCV (Mean corpuscular volume) | 1.88ⅹ10^-1^ | 96.68 |
| 64 | APTT (Activation of partial thromboplastin time) | 2.40ⅹ10^-1^ | 72.02 |
| 65 | pH | 2.69ⅹ10^-1^ | 45.15 |
| 66 | Quantification of Treponema pallidum antibodies | 3.09ⅹ10^-1^ | 62.33 |
| 67 | Hemoglobin | 3.50ⅹ10^-1^ | 96.68 |
| 68 | Chloride | 3.68ⅹ10^-1^ | 95.29 |
| 69 | HIV antibody quantification | 4.49ⅹ10^-1^ | 62.05 |
| 70 | Hct (Hematocrit) | 4.88ⅹ10^-1^ | 96.68 |
| 71 | Monocytes count | 5.04ⅹ10^-1^ | 96.68 |
| 72 | Fibrinogen | 6.59ⅹ10^-1^ | 72.02 |
| 73 | IL-1β (Interleukin 1β) | 8.87ⅹ10^-1^ | 16.07 |


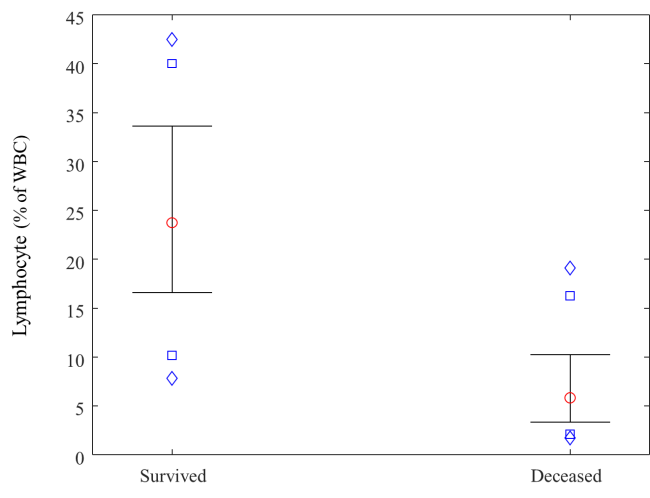

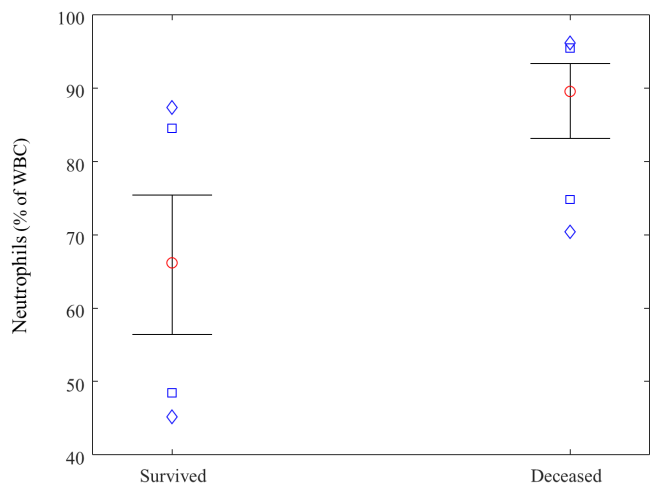


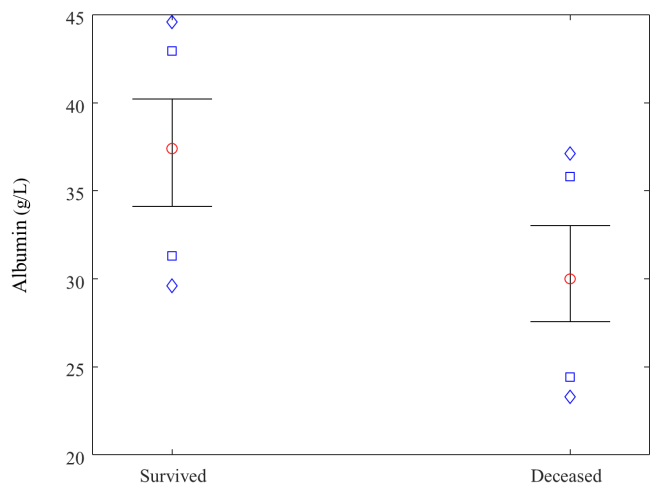

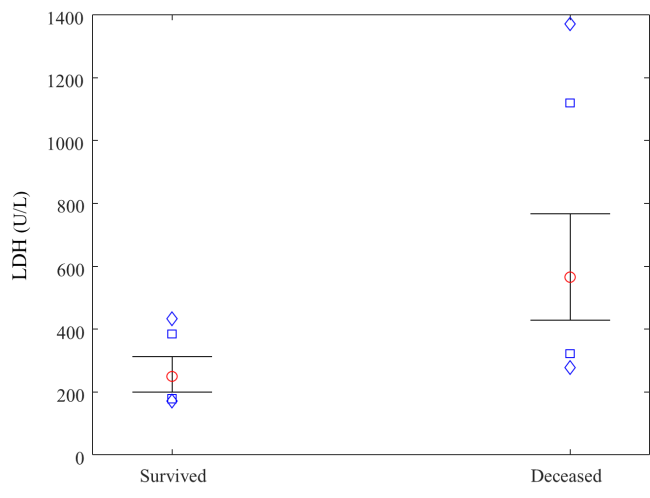


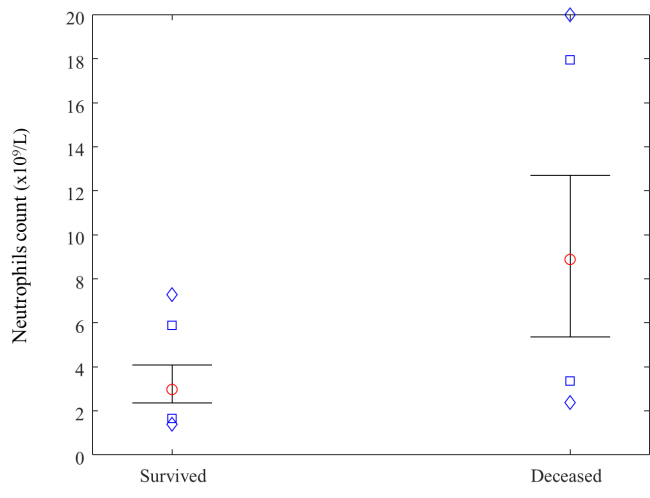

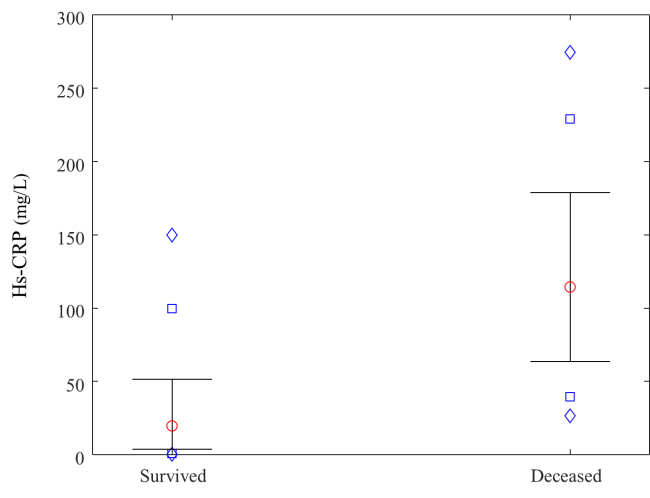


Figure S1. (Continued) Sample distributions of the selected 28 biomarkers in the survival and deceased groups


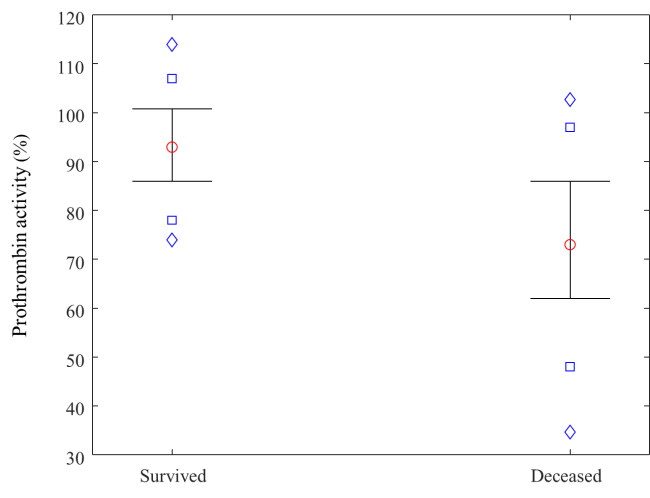

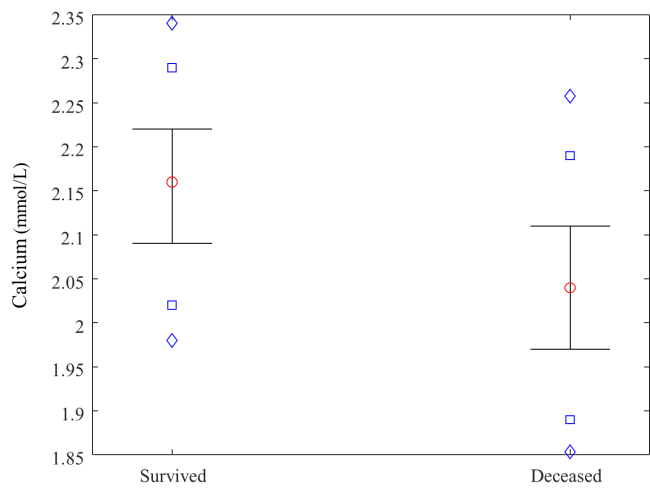


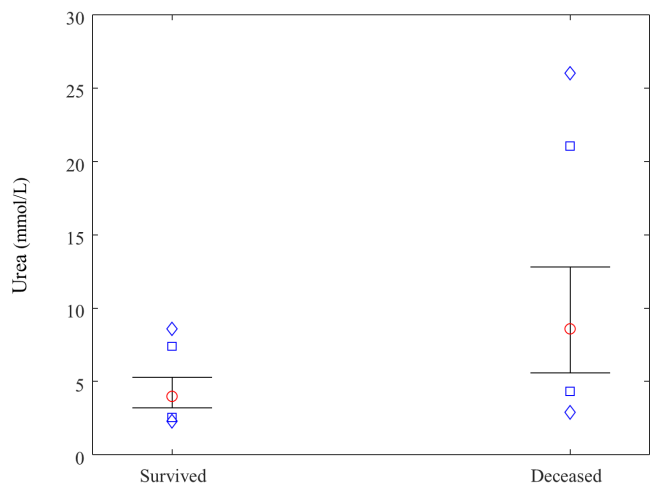

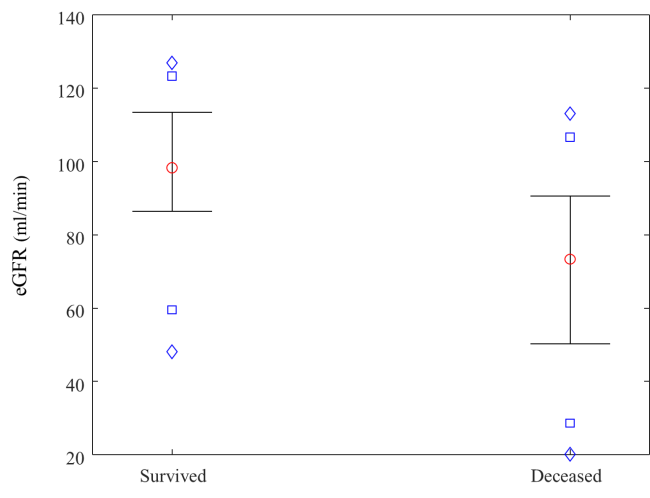


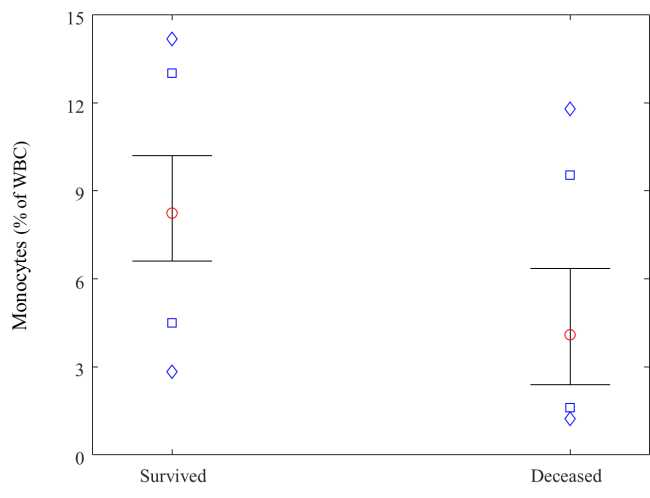

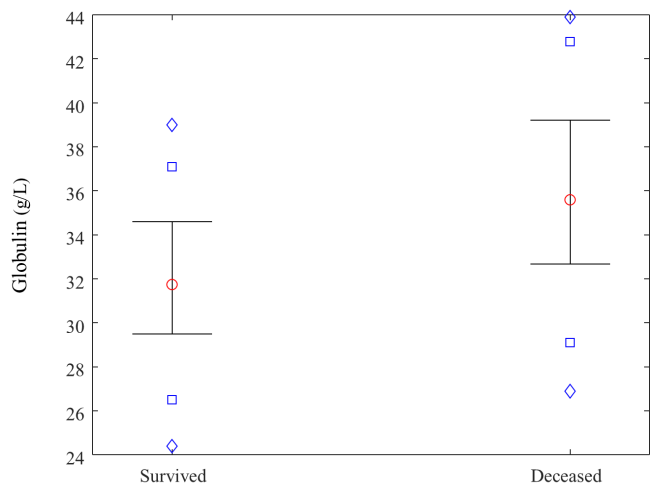


Figure S1. (Continued) Sample distributions of the selected 28 biomarkers in the survival and deceased groups


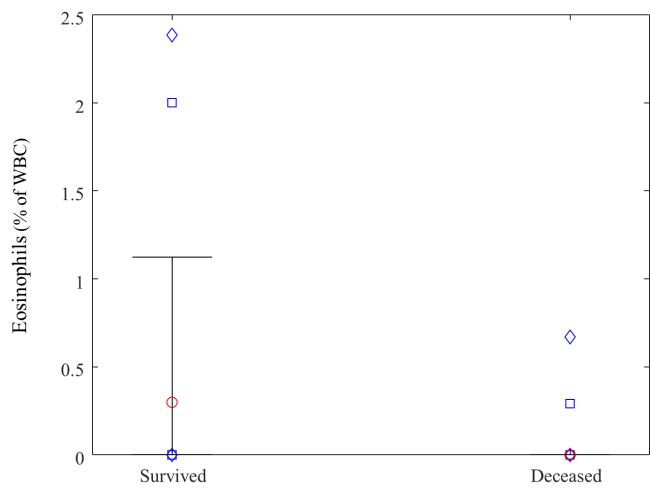

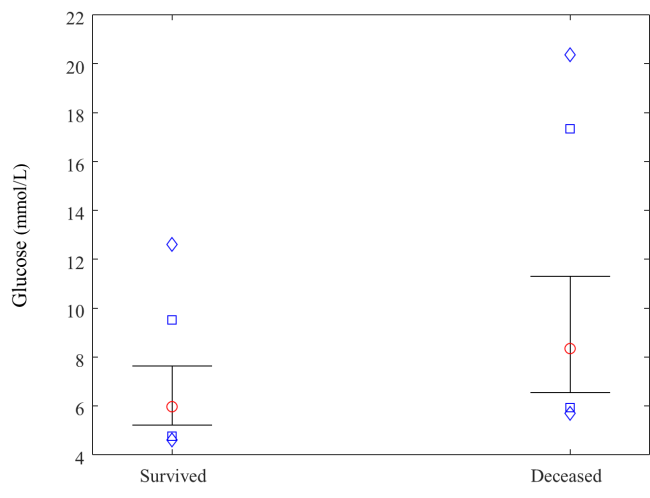


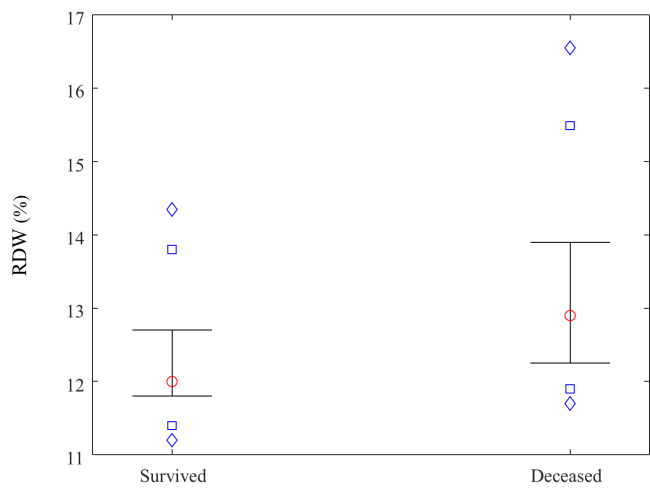

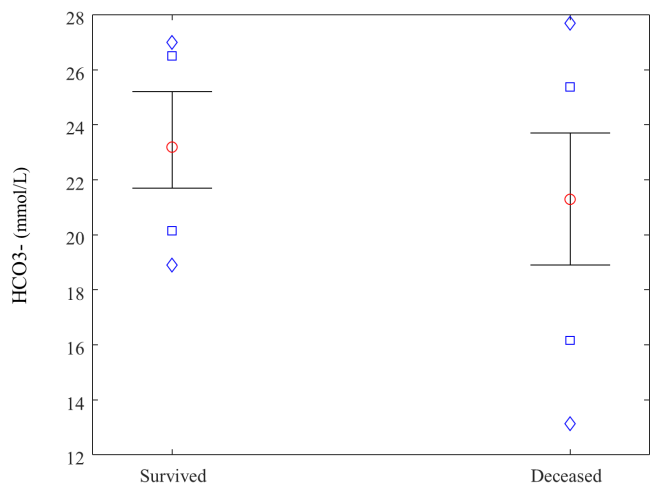


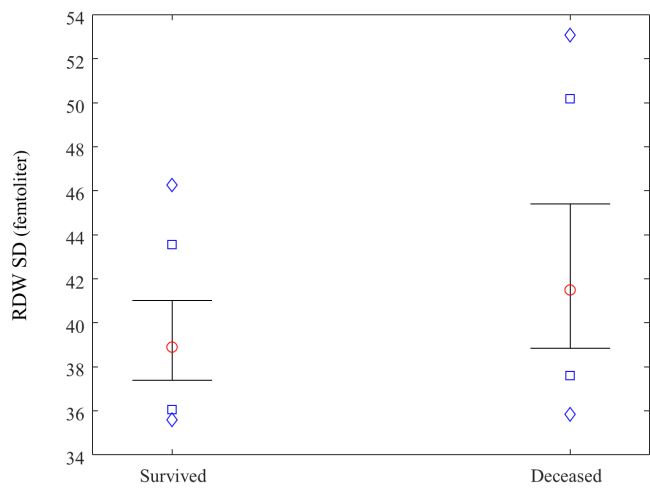

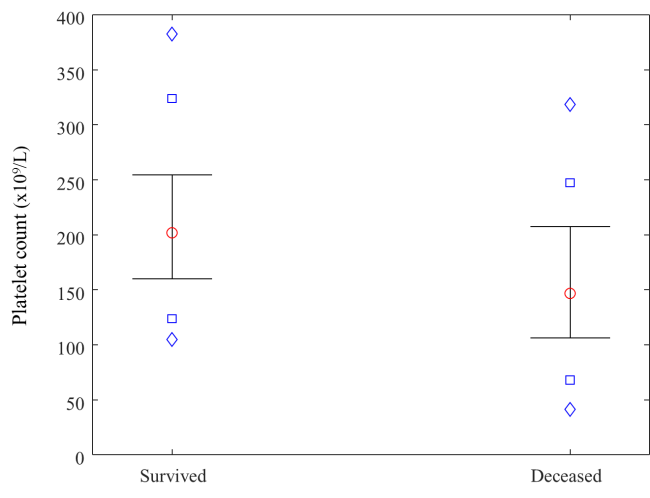


Figure S1. (Continued) Sample distributions of the selected 28 biomarkers in the survival and deceased groups


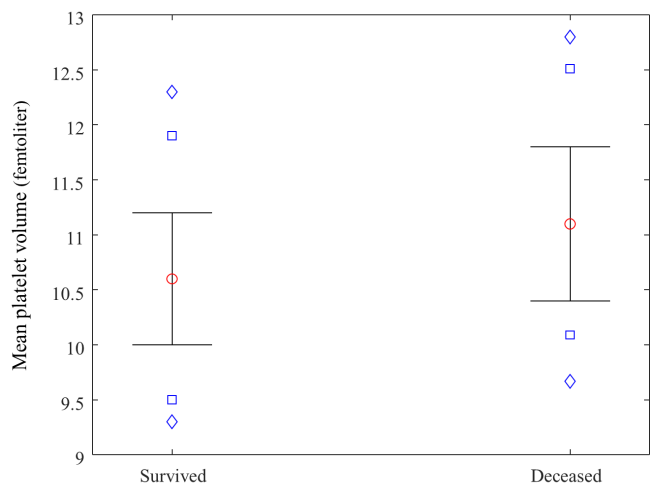

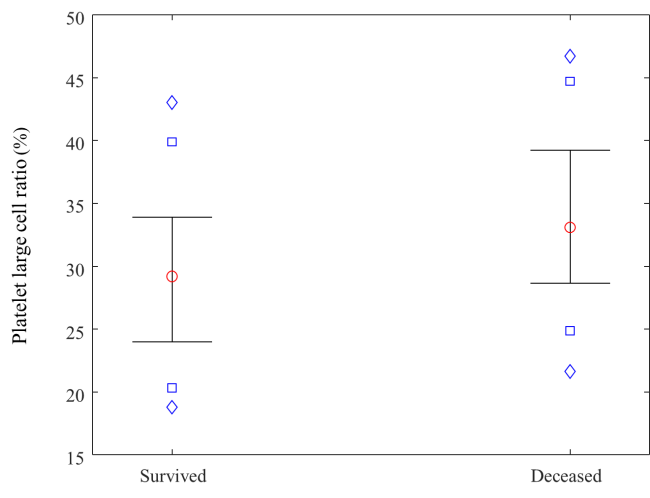

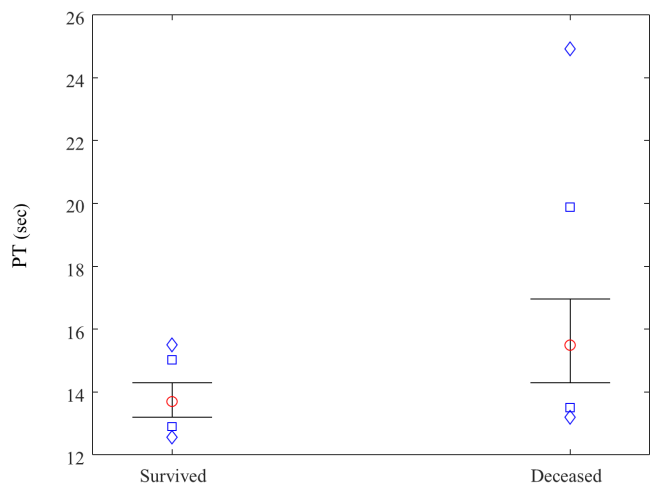

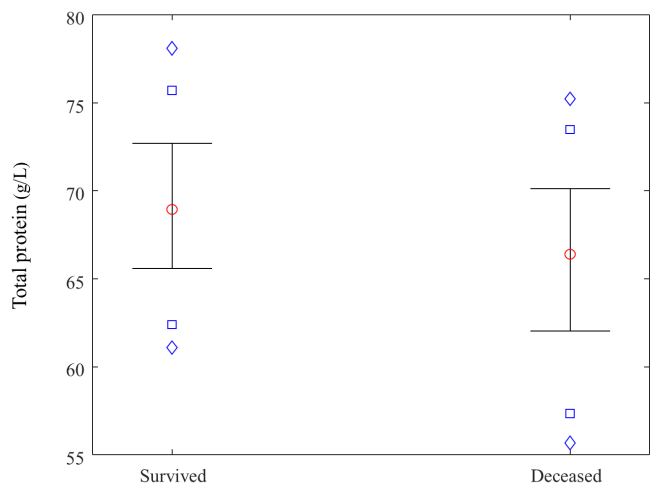


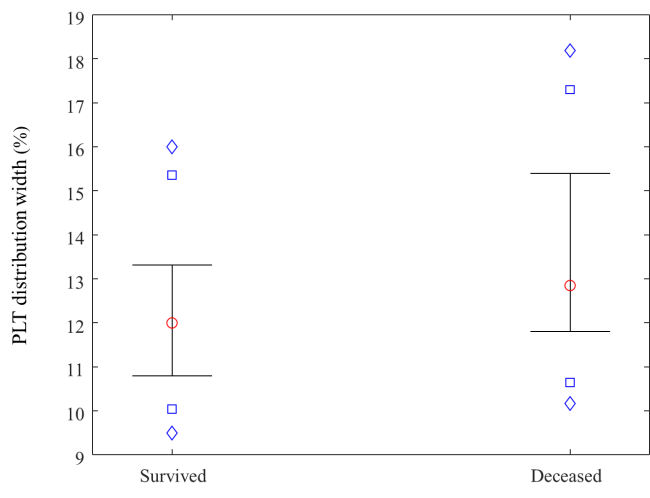

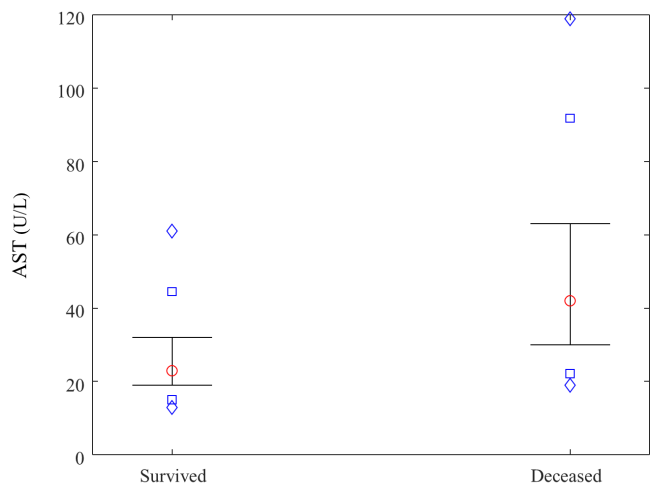


Figure S1. (Continued) Sample distributions of the selected 28 biomarkers in the survival and deceased groups


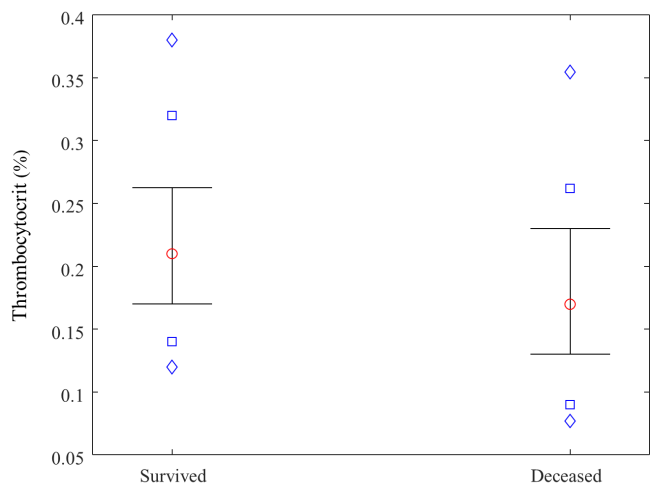

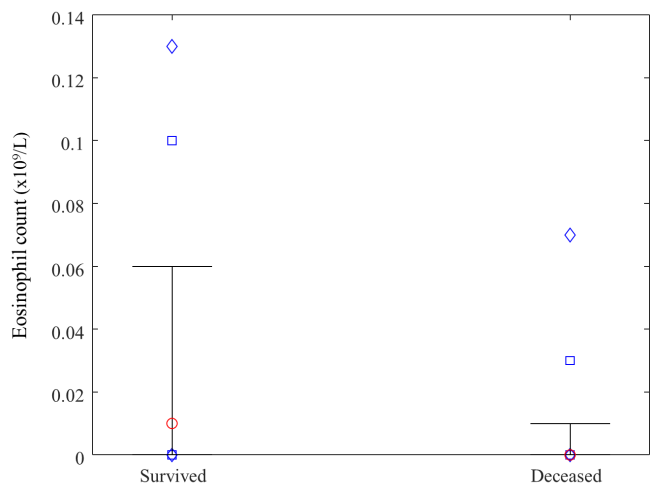


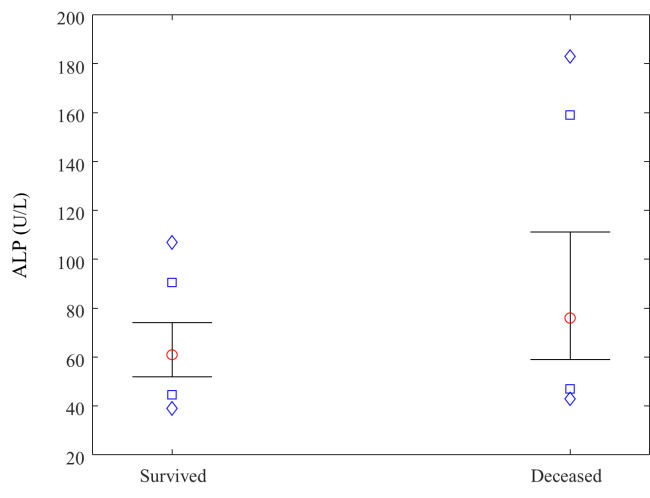

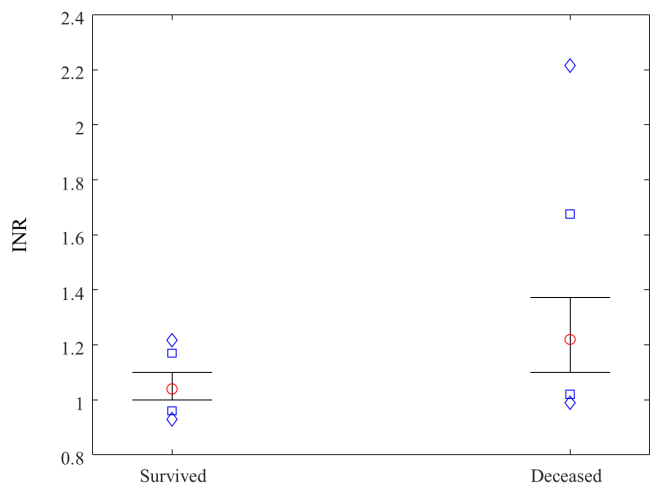


Figure S1. Sample distributions of the selected 28 biomarkers in the survival and deceased groups


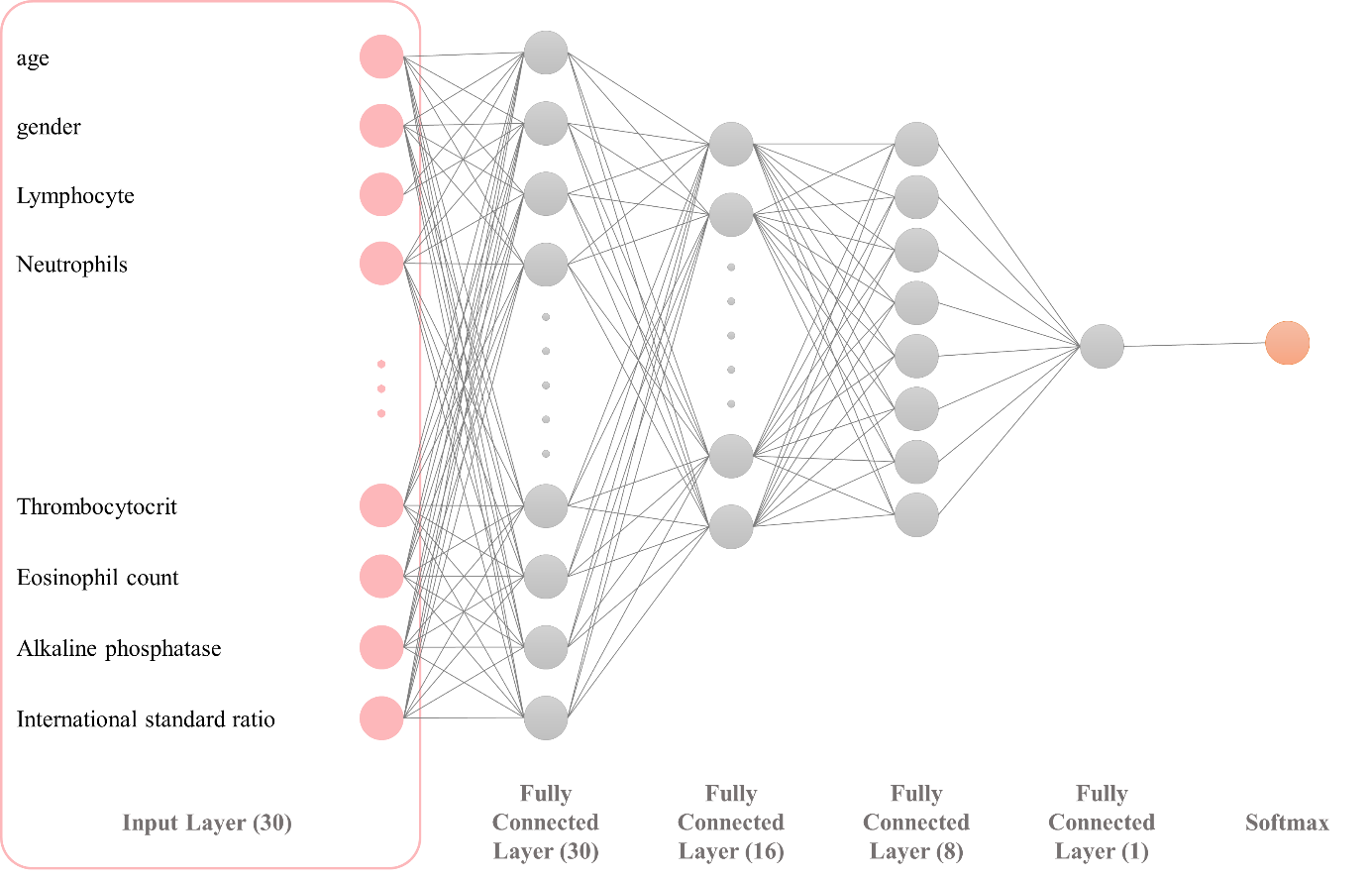


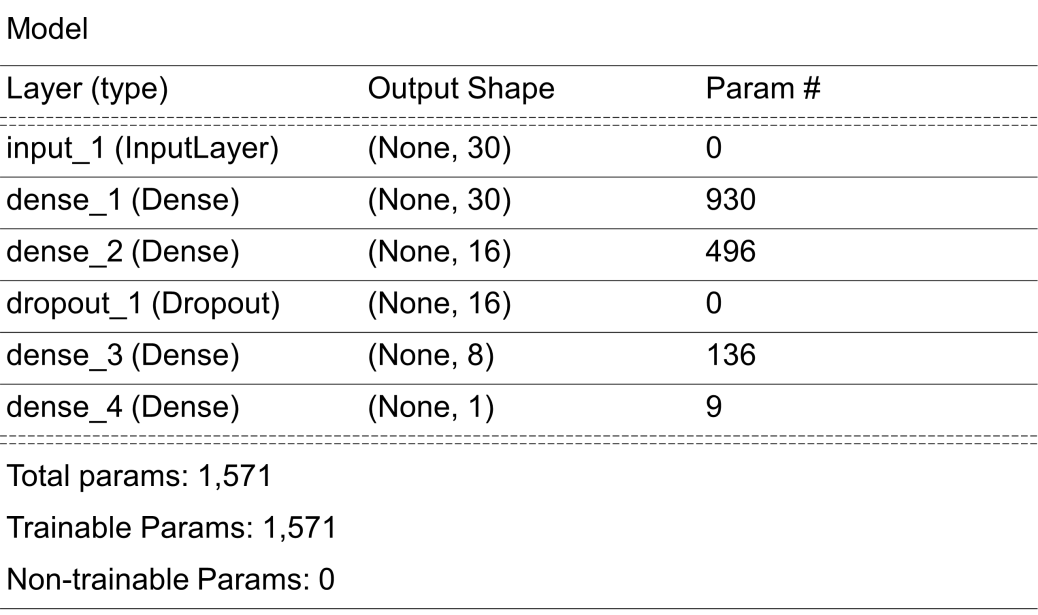


Figure S2. (Top) Our DNN model and (Bottom) the printed textual summary run on Keras.

Table S2. Optimal hyper-parameters of random forest, XGBoost (XGB) and AdaBoost (AB) models

| **hyper-parameter** | **Random Forest** | **XGBoost** | **AdaBoost** |
| --- | --- | --- | --- |
| Maximum depth of the tree | 4 | 2 |  |
| Maximum number of the features | 5 |  |  |
| Number of estimator | 100 | 100 | 100 |
| Kernel |  |  |  |
| C |  |  |  |
| Gamma |  |  |  |
| Objective |  | Logistic |  |
| Learning rate |  | 10.0 | 0.1 |
